# Overexpression of a “*Candidatus* Liberibacter Asiaticus” Effector Gene *CaLasSDE115* Contributes to Early Colonization in *Citrus sinensis*

**DOI:** 10.3389/fmicb.2021.797841

**Published:** 2022-02-21

**Authors:** Meixia Du, Shuai Wang, Liting Dong, Rongrong Qu, Lin Zheng, Yongrui He, Shanchun Chen, Xiuping Zou

**Affiliations:** National Citrus Engineering Research Center, Citrus Research Institute, Southwest University, Chongqing, China

**Keywords:** Citrus Huanglongbing, *Candidatus* Liberibacter asiaticus, effector, LasSDE115, overexpression, SAR, early colonization

## Abstract

Huanglongbing (HLB), caused by “*Candidatus* liberibacter asiaticus” (*Ca*Las), is one of the most devastating diseases in citrus but its pathogenesis remains poorly understood. Here, we reported the role of the *Ca*LasSDE115 (CLIBASIA_05115) effector, encoded by *Ca*Las, during pathogen-host interactions. Bioinformatics analyses showed that *Ca*LasSDE115 was 100% conserved in all reported *Ca*Las strains but had sequence differences compared with orthologs from other “*Candidatus* liberibacter.” Prediction of protein structures suggested that the crystal structure of *Ca*LasSDE115 was very close to that of the invasion-related protein B (IalB), a virulence factor from *Bartonella henselae*. Alkaline phosphatase (PhoA) assay in *E. coli* further confirmed that *Ca*LasSDE115 was a Sec-dependent secretory protein while subcellular localization analyses in tobacco showed that the mature protein of SDE115 (mSDE115), without its putative Sec-dependent signal peptide, was distributed in the cytoplasm and the nucleus. Expression levels of *CaLasSDE115* in *Ca*Las-infected Asian citrus psyllid (ACP) were much higher (∼45-fold) than those in *Ca*Las-infected Wanjincheng oranges, with the expression in symptomatic leaves being significantly higher than that in asymptomatic ones. Additionally, the overexpression of *mSDE115* favored *Ca*Las proliferation during the early stages (2 months) of infection while promoting the development of symptoms. Hormone content and gene expression analysis of transgenic plants also suggested that overexpressing *mSDE115* modulated the transcriptional regulation of genes involved in systemic acquired resistance (SAR) response. Overall, our data indicated that *Ca*LasSDE115 effector contributed to the early colonization of citrus by the pathogen and worsened the occurrence of Huanglongbing symptoms, thereby providing a theoretical basis for further exploring the pathogenic mechanisms of Huanglongbing disease in citrus.

## Introduction

Citrus Huanglongbing (HLB) is the most devastating disease which has harmed citrus trees in more than 50 countries and regions around the world, thereby causing serious economic losses to the citrus industry (Gottwald, 2010). Its causative agent is a phloem-limited α-*Proteobacterium* “*Candidatus* Liberibacter” (Jagoueix et al., 1994; Bové, 2006) for which three species namely “*Candidatus* Liberibacter asiaticus (*Ca*Las),” “*Candidatus* Liberibacter africanus” and “*Candidatus* Liberibacter americanus” are known to be associated with HLB in citrus (Teixeira et al., 2005; Wulff et al., 2014; Lin et al., 2015). However, to date, HLB pathogens have not been successfully cultured *in vitro*. Among these, *Ca*Las is the most prevalent one in citrus, with its insect vector being the Asian citrus psyllid (ACP) *Diaphorina citri* Kuwayama. *Ca*Las resides in sieve elements and spreads through the phloem transport system (Duan et al., 2009). HLB symptoms mainly include flush yellowing, leaf mottling, as well as inverted fruit coloring before eventually resulting in plant death (Bassanezi et al., 2009; Graham et al., 2013). Although there have been many successful cases of HLB management based on the “Three basic measures” (planting HLB-free nursery trees, timely clearing of any HLB-infected trees and controlling psyllid populations as much as possible), neither effective cures against the disease and nor HLB-resistant citrus cultivars have, so far, been identified (Wang et al., 2017).

Host-pathogen interactions are vital to understanding and controlling HLB disease. This interplay between hosts and pathogens involves a myriad of molecular interactions that play important roles both in a pathogen’s ability to suppress or avoid a plant’s immunity as well as in the host’s ability to detect and remove the pathogen. First, the plant perceives pathogen-associated molecular patterns (PAMP) such as fungal chitin and bacterial flagellin through its immune receptors PRRs (pattern recognition receptors) to initiate PAMP-triggered immunity (PTI) that prevents pathogen invasion (Boller and Felix, 2009; Segonzac and Zipfel, 2011). To defeat the PTI responses, pathogens manipulate the host’s cellular functions by secreting diverse effector proteins (Yuan et al., 2021) which are recognized by specialized receptors (usually the plant disease resistance proteins) and this further leads to the activation of the host’s effector-triggered immunity (ETI) to prevent pathogen attack (Jones and Dangl, 2006; Haitao et al., 2015). However, a pathogen can also evolve its effectors to suppress the ETI (Nguyen et al., 2021). In *Ca*Las-citrus interactions, the effectors involved and their functions in pathogen virulence are poorly understood, partly due to the unculturability of the pathogen. Thus, exploring the role of *Ca*Las effectors could be key to understanding the pathogenicity mechanisms of *Ca*Las as well as for identifying citrus resistance genes which could eventually be exploited for developing new control methods and improving HLB resistance in citrus.

Many extracellular plant pathogens suppress plant immunity through the translocation of effector proteins using the type-III secretion system (T3SS) (Jones and Dangl, 2006; Toruno et al., 2016). However, since *Ca*Las does not possess a T3SS (Duan et al., 2009) but instead harbors a complete Sec-dependent secretion system (Duan et al., 2009), it is believed that *Ca*Las secrets Sec-dependent effectors (SDEs) including virulence factors into plant cells through this system (Pitino et al., 2016; Prasad et al., 2016; Pagliaccia et al., 2017). In other phloem-limited pathogens such as phytoplasma, several SDEs have been shown to be critical for pathogenicity (Saskia et al., 2008). Similarly, for *Ca*Las SDEs, 86 putative SDE proteins have been shown to have functional Sec-dependent secretion signal peptides (Prasad et al., 2016). Clark et al. (2018) reported that *Ca*Las SDE1 suppressed citrus immunity by inhibiting the activity of papain-like cysteine proteases (PLCPs). Pang et al. (2020) showed that SDE15 suppressed plant immunity by interacting with the citrus protein CsACD2, a homolog of *Arabidopsis* ACCELERATED CELL DEATH 2. In fact, overexpression of *SDE15* or *CsACD2* in Duncan grapefruit was found to suppress plant immunity and promote *Ca*Las growth. Furthermore, the authors indicated that SDE15-CsACD2 interactions repressed citrus PTI by affecting the expression of PTI marker genes such as *FRK1*, *GST1* and *WRKY22*. Other *Ca*Las SDEs were also reported to regulate plant immunity responses (Liu et al., 2019; Ying et al., 2019; Li et al., 2020). In addition, it has been shown that *Ca*Las also transported non-classically secreted proteins (ncSecPs) into host cells to regulate host immunity. In this context, Du et al. (2021) indicated that 10 ncSecPs had opposing effects on early plant defenses. For instance, SC2_gp095 effectors, encoded by *Ca*Las prophages, could suppress the development of host symptoms through the manipulation of plant H_2_O_2_-mediated defense signaling (Jain et al., 2015). Similarly, *Ca*Las SahA could degrade salicylic acid (SA) to suppress plant defenses (Li et al., 2017) while the peroxiredoxin effector LasBCP simultaneously inhibited localized as well as systemic innate immune responses through oxylipin-mediated defense signaling in plants (Jain et al., 2018, 2019).

In this study, we investigated the potential role of a putative virulence factor *Ca*LasSDE115 (CLIBASIA_05115) during *Ca*Las infections. Bioinformatics predictions indicated that *Ca*LasSDE115 was very close to that of a virulence factor IalB from *Bartonella henselae*, which is not only an invasion-associated locus B but is also involved in pathogen-host invasions (Vayssier-Taussat et al., 2010). The sec-dependent secretory characteristics of *Ca*LasSDE115 was also confirmed by bioinformatics analyses and phoA assays while its involvement in citrus’ responses to *Ca*Las infections were investigated by overexpressing *mSDE115* in HLB-susceptible Wanjincheng oranges. Altogether, our data indicated that *Ca*LasSDE115 positively regulated early pathogenic colonization of citrus by modulating the transcriptional regulation of genes involved in SAR responses. The potential mechanisms of *Ca*LasSDE115 were eventually discussed in this study. Overall, it is expected that this study will help to further reveal the pathogenic mechanisms of Huanglongbing disease in the future.

## Materials and Methods

### Plant Materials, Asian Citrus Psyllids, Pathogens and Growth Conditions

Wanjincheng orange plants (*C. sinensis* Osbeck) as well as all the transgenic ones used in this study were plantged in a greenhouse, with a 16 h photoperiod of 45 μmol m^–2^s^–1^ illumination and a relative humidity of 60%, at the National Citrus Engineering Research Center, Chongqing, China. Citrus trees containing *Ca*Las were also maintained in the greenhouse. Healthy adult ACPs were reared on young leaf flush of *Ca*Las-infected or *Ca*Las-free sweet orange (*C. sinensis*) seedlings in incubators at a temperature of 25 ± 1°C and after 3-weeks of feeding, the insects were collected, frozen with liquid nitrogen and stored at -80°C. Using the primers Cs16S-F/16s-R (Supplementary Table 1), the presence of the *Ca*Las pathogens in both plants and ACPs was confirmed by PCR.

### Cloning and Analysis of *CaLasSDE115*

Using cDNA from *Ca*Las-infected Wanjincheng orange plants as a template, the gene encoding for *Ca*LasSDE115 (CLIBASIA_05115) was amplified with the primer pair T-SDE115-F/R (Supplementary Table 1). The PCR was performed in a final volume of 50 μL, containing 25 μL of Primer STAR Max Premix (TaKaRa, Ojin, Japan), 1.5 μL of each primer (10 mM⋅L^–1^), 19 μL of H_2_O and 3 μL of cDNA (0.5 × 10^–5^ ng⋅L^–1^). The PCR conditions were as follows: pre-denaturation at 96°C for 5 min, followed by 35 amplification cycles (96°C for 30 s, 56°C for 30 s and 72°C for 30 s), before a final extension at 72°C for 3 min. The PCR product was T-cloned into the pGEM-Teasy vector (Promega, WI, United States). The sequence of *CaLasSDE115* was then determined by Sanger sequencing.

The signal peptide (SP) and transmembrane structure of *Ca*LasSDE115 were predicted using the signalP5.0 tool^1^ and TMHMM Server (v.2.0)^2^, respectively. The secondary as well as tertiary structures of its mature protein were analyzed using SOPMA^3^, SWISS-MODEL^4^ and Phyre^5^ tools, respectively. Multiple sequence alignment of the *Ca*LasSDE115 protein with homologs from other selected species was also performed using ClustalW2 tool^6^. A phylogenetic tree was constructed based on the neighbor-joining method using MEGA7.0 software (Kumar et al., 2016).

### Alkaline Phosphatase (PhoA) Assay

PhoA assays of *Ca*LasSDE115 were performed, in triplicate, as previously described (Liu et al., 2019). *CaLasSDE115*, *mSDE115* without the SP-coding sequence, as well as *SDE115sp* containing only the SP-coding sequences were separately inserted into *mphoA*-containing pET-mphoA (without the SP-coding sequence), to generate different in-frame gene fusions while PET-phoA (containing its SP-coding sequence) and PET-mphoA vectors were used as positive and negative controls, respectively. These different constructs were then transformed into *E. coli* BL21 cells which were incubated overnight at 37°C on LB medium supplemented with 90 μg/ml of BCIP (5-bromo-4-chloro-3-indolylphosphate) and 75 mM of Na_2_HPO_4_ in order to detect phoA activities. The blue transformants indicated that fusion proteins were secreted outside the cells.

### Subcellular Localization Analysis in *Nicotiana benthamiana* Cells

The *mSDE115* sequence was cloned into pCAMBIA1300-35S-GFP to construct the fusion gene *mSDE115:GFP* prior to its transfer into *A. tumefaciens* EHA105. Agroinfiltration of *N. benthamiana* leaves was performed as described before (Liu et al., 2019) and 3 days post-inoculation, the localization of the fusion protein was determined using an FV3000 confocal microscope equipped with a UV light source (Olympus, Tokyo, Japan). A histone 2B (H2B) fusion with the red fluorescent protein (RFP) was used as a marker for the nucleus (Martin et al., 2009). This test was repeated twice.

### Citrus Transformation

For citrus transformation, the *mSDE115* gene sequence was first cloned into a pLGN vector (Zou et al., 2017) to construct the *p35S:mSDE115* vector in which *mSDE115* expression was controlled by a 35S strong constitutive promoter. The vector was then introduced into an EHA105 strain.

Epicotyl segments from Wanjincheng orange seedlings were used for *A. tumefaciens*-mediated transformation, with the experiment performed as previously described (Peng et al., 2015). Transgenic shoots were then identified by GUS staining as well as PCR analysis before being micrografted onto Troyer citrange [*Poncirus trifoliata* (L.) Raf. × *C. sinensis* (L.) Osbeck] seedlings *in vitro*. The resulting plantlets were further grafted onto Troyer citrange seedlings in a greenhouse, and the expression levels of *mSDE115* gene in those transgenic plants were determined by RT-qPCR analysis.

### Evaluation of Huanglongbing Tolerance in Transgenic Citrus

Evaluating the tolerance of transgenic plants to *Ca*Las was performed as previously described by Zou et al. (2017). Three to five plants per independent transgenic line, including wild type (WT) plants, were inoculated by *Ca*Las-infected Wanjincheng orange branches using the shoot top grafting method (Cifuentes-Arenas et al., 2019). All the inoculated plants were then maintained in a greenhouse and regularly checked for any sign of disease incidence.

The *Ca*Las bacterial populations (*Ca*Las cells μg^–1^ of citrus DNA) were determined as previously described (Zou et al., 2017). Every 2 months, three midrib sections from three different leaves per plant were randomly extracted and pooled into one sample from which DNA was isolated. The amount of *Ca*Las 16S rRNA genes and citrus 18S rRNA genes in the isolated DNA samples were subsequently detected by quantitative PCR (qPCR) using the Cs16S-f/Cs16S-r and Cs18S-f/Cs18S-r primers (Supplementary Table 1), respectively. The qPCR was performed in a final volume of 12 μL, containing 6 μL of the SYBRPRIME qPCR Kit (Bioground Biotech, Chongqing, China), 4.4 μL H_2_O, 0.3 μL of each primer (10 mM⋅L^–1^) as well as 1 μL DNA(10 ng⋅μL^–1^)while the PCR protocol involved a pretreatment (95°C for 2 min) followed by 40 amplification cycles (each at 94°C for 10 s and 60°C for 60 s). Using the citrus 18S gene as the internal reference, the *Ca*Las bacterial populations were calculated as reported by Zou et al. (2017). In this case, the disease intensity in the transgenic lines was evaluated based on the bacterial populations of three to five plants compared with the WT control.

### RT-qPCR Analysis

Total RNA isolation from citrus tissues, including leaves, roots and midrib sections were performed using the EASYspin Plant RNA Extraction Kit according to the manufacturer’s instructions (Aidlab, Beijing, China). In the case of ACPs, 30–40 *Ca*Las-infected insects were ground to a powder in liquid nitrogen before immediately extracting total RNA with TRIzol Reagent (Invitrogen, MA, United States). A 1 μg cDNA was then synthesized by reverse transcription (RT) using the PrimeScript ™RT Master Mix (TaKaRa, Ojin, Japan) prior to quantitative reverse transcription-polymerase chain reaction (QRT-PCR using the primers listed in Supplementary Table 1). Gene expression was detected using the SYBRPRIME qPCR Kit (Bioground Biotech, Chongqing, China). The PCR reactions were as follows: pre-denaturation (50°C for 60 s, 95°C for 120 s), followed by 40 cycles of amplification (each at 95°C for 5 s, 60°C for 15 s). The *Ca*Las DNA *gyrase subunit A* (*CaLasgyrA*, GenBank No. CP001677.5) gene was employed as an internal reference to investigate the expression of *CaLas05115* in *Ca*Las-infected plants and ACPs while the citrus *GAPDH* gene (Mafra et al., 2012) was used as an internal reference to evaluate gene expression in transgenic plants. All experiments were performed in triplicate. All the relative expression values were calculated by the Ct method (2^–Δ^ ^Δ^ *^Ct^*) (Livak and Schmittgen, 2001).

### Measurement of Hormone Content

Fresh tissues (0.1 g) were collected from three fully mature leaves per line and ground to a fine powder in liquid nitrogen. The powder was then homogenized in 0.9 mL of phosphate saline buffer (PBS) of pH = 7.4, before being centrifuged at 800 rpm for 10 min. The resulting supernatant was transferred to a new, enzyme-free tube and stored in -20°C until assayed.

The SA, methyl salicylate (MeSA) and jasmonic acid (JA) content in the supernatant was determined using plant enzyme-linked immunosorbent assay (ELISA) kits (Jiweibio, Shanghai, China) (Zou et al., 2021). Briefly, the supernatant was first diluted with a 5 × ELISA diluent buffer before adding 50 μL of the diluted supernatant to reaction wells, followed by 100 μl of the detection antibody (horseradish peroxidase-conjugated antibody). After incubating the mixture at 37°C for 1 h, the wells were washed five times with 350 μL of the washing buffer prior to a second incubation with 100 μL of 1 × TMB (3,3′, 5,5′-tetramethylbenzidine) solution at 37°C for 15 min. The reaction was eventually stopped by adding 50 μL of the stop solution. The OD of each well was then measured, at 450 nm, using a Spectra-Max^®^ M2 microplate reader (Molecular Devices Corporation, Menlo Park, CA, United States). The hormone content per gram of fresh weight leaf (μg/g) was calculated using Excel 365. All the assays were repeated three times.

### Statistical Analyses

Statistical analyses of all data were conducted in Excel 365, using the Student’s *t*-test to compare differences between the control and samples at 5% significance level.

## Results

### Cloning and Sequence Analysis of *CaLasSDE115*

**FIGURE 1:**
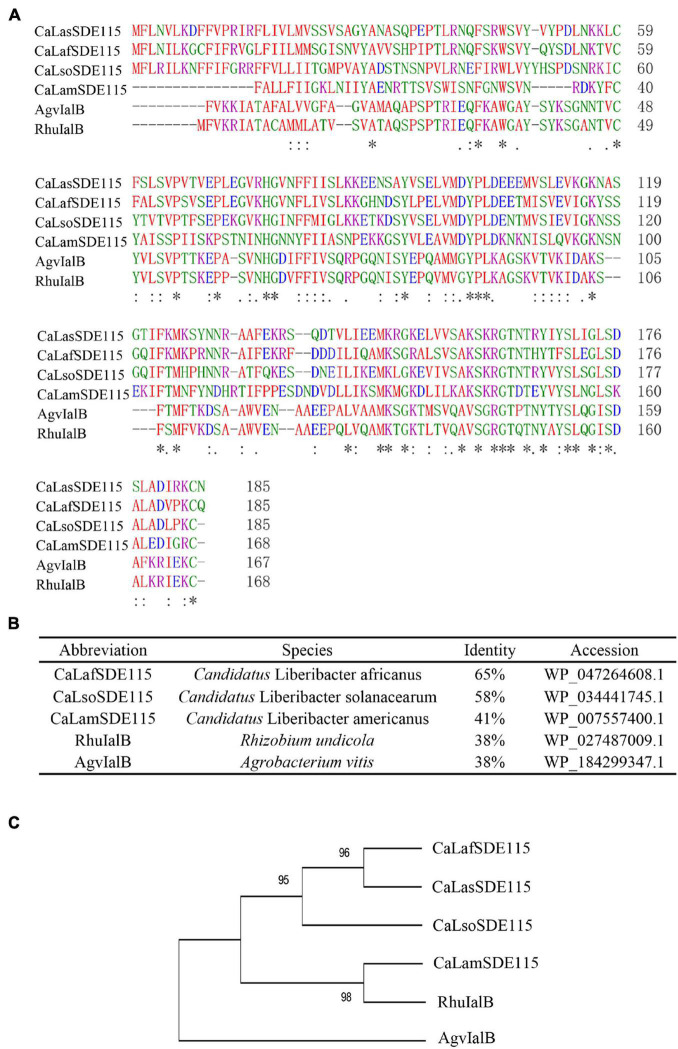
Analysis of *Ca*LasSDE115 protein sequence. **(A,B)** Multiple sequence alignment of *Ca*LasSDE115 with selected homologs. **(C)** A phylogenetic tree containing *Ca*LasSDE115 and other selected homologs. The reliability of the tree was estimated using 1000 bootstrap replicates and the bootstrap values (%) are shown above branches. In **(A)**, “*”, “:” and “.” indicate a single completely conservative, highly and low similar among residues, respectively.

Based on the sequence of *CaLasSDE115* from *Ca*Las psy62 (Duan et al., 2009), a 558-bp long coding sequence was cloned from *Ca*Las-infected Wanjincheng oranges. This gene encoded 186 amino acid residues and was found to be 100% conserved in all *Ca*Las strains based on the complete genomes which are currently available (Supplementary Figure 1). Multiple alignments were performed by ClustalW2 using *Ca*LasSDE115 homologs from selected, different species (Figures 1A,B). The results showed that *Ca*LasSDE115 had a 41 to 65% similarity in amino acid sequence compared with the selected orthologs from other *Candidatus* Liberibacters. Phylogenetic analysis also showed that *Ca*LasSDE115 was closely related to the orthologs from “*Candidatus* Liberibacter africanus” and “*Candidatus* Liberibacter solanacearum” (Figure 1C). The prediction of tertiary structures further indicated that the crystal structure of *Ca*LasSDE115 was very close to that of the virulence factor IalB from *Bartonella henselae* (Vayssier-Taussat et al., 2010) despite only 15.6% of sequence identity between them (Supplementary Figures 2, 3). In fact, the amino acid sequence of *Ca*LasSDE115 had a 38% identity to those of the IalB homologs from *Rhizobium undicola* and *Agrobacterium vitis* (Figure 1B). These results suggested that *Ca*LasSDE115 was involved in *Ca*Las pathogenicity.

### *Ca*LasSDE115 as a Sec-Dependent Presecretory Protein

**FIGURE 2:**
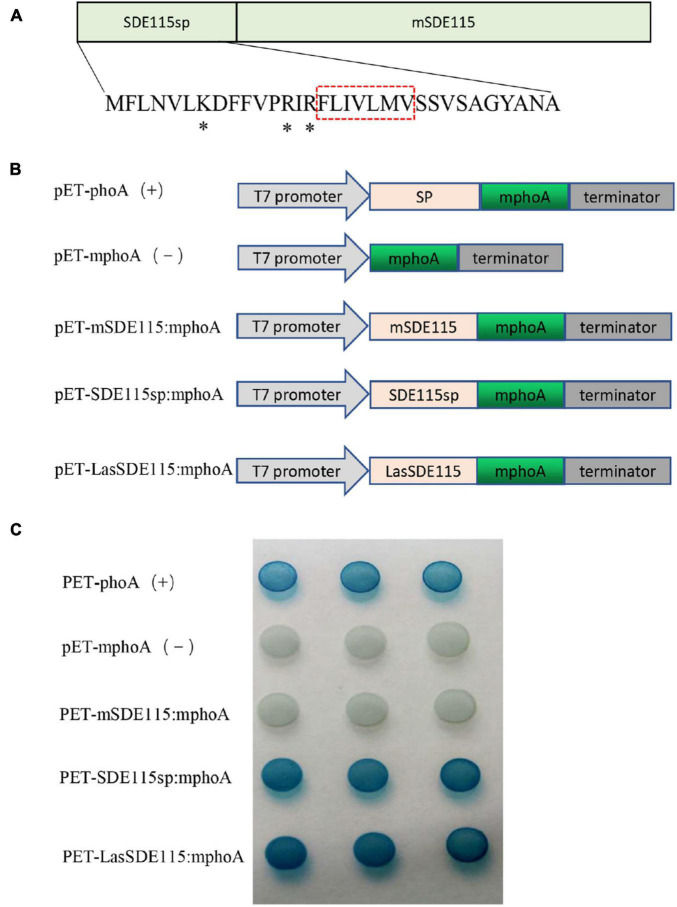
*Ca*LasSDE115 is a Sec-dependent secretory protein. **(A)** Structure of the *Ca*LasSDE115 protein. Its signal peptide and mature protein were named SDE115sp and mSDE115, respectively. *indicated the positively charged amino acids lysine (K) and arginine (R) while the red box indicated the central hydrophobic core. **(B)** Schematic diagram of the prokaryotic expression cassettes for phoA assay. **(C)** PhoA assay for the secretion of *Ca*LasSDE115 in *E. coli* cells. The data showed that SDE115sp directed the extracellular translocation of mPhoA lacking its native SP and that of *Ca*LasSDE115:mphoA fusion.

SignalP5.0 predicted that *Ca*LasSDE115 was a Sec-dependent presecretory protein containing a putative signal peptide (namely SDE115sp) with a cleavage site between amino acids 32 and 33 (ANA-SQ; probability: 0.885) (Figure 2A). The SDE115sp had a hydrophobic core, flanked by three positively charged residues at the N-terminus and a less positively charged C terminus (Figure 2A), which had the characteristics of a Sec-dependent signal peptide (Tamar and Damon, 2018). Based on the TMHMM Server (v.2.0) analysis, no transmembrane domains were found in the mature *Ca*LasSDE115 protein. To determine the export of this protein via the Sec-dependent secretory system, an *E. coli* phoA assay was also used (Figures 2B,C). In this case, the data showed that bacteria containing fusions between mphoA (without its native SP) and either the native *Ca*LasSDE115 or only its signal peptide SDE115sp became blue overnight. On the other hand, bacteria containing only mphoA (without its native SP) or its fusion with the mature protein mSDE115 remained white. These results, along with the positive control containing the complete phoA (mphoA + its native SP), confirmed that *Ca*LasSDE115 was a typical Sec-dependent secretory protein and that the signal peptide, as predicted by SignalP5.0, was involved in directing extracellular secretion of proteins in bacteria.

### Subcellular Localization of the *Ca*LasSDE115 Mature Protein in Tobacco Cells

**FIGURE 3:**
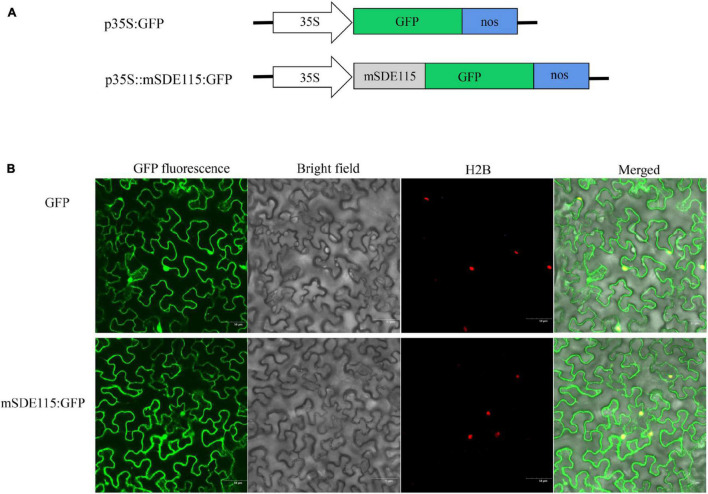
Subcellular location of mSDE115 in tobacco epidermis cell. **(A)** Schematic diagram of the constructs used for agroinfiltration. A 35S, CaMV 35S promoter; NOS, the nopaline synthase terminator. **(B)** Subcellular localization of mSDE115:GFP fusion protein as observed by confocal microscopy. A histone 2B (H2B) fusion with the red fluorescent protein (RFP) was used as a marker for the nucleus (Martin et al., 2009).

Since the above results revealed that the *Ca*LasSDE115 mature protein crossed the bacterial outer membrane into the host cells via a Sec-dependent secretory system, the next step was to observe the subcellular localization of *Ca*LasSDE115 mature proteins in plants. For this purpose, the mSDE115 coding sequence was fused with the GFP reporter gene (Figure 3A). Transient expression tests in *N. benthamiana* leaves showed that the mSDE115:GFP fusion had similar cellular locations as those of the GFP protein alone, with fluorescence signals detected in the nucleus and cytoplasm (Figure 3B).

### Transcription Characteristics of *CaLasSDE115* in Wanjincheng Oranges and Psyllids

**FIGURE 4:**
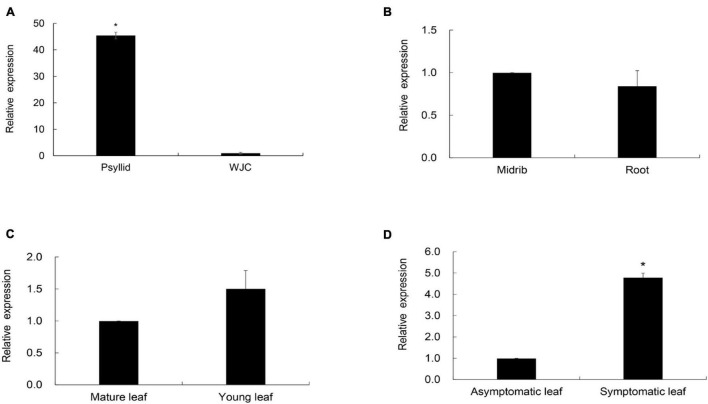
Transcription levels of *CaLasSDE115* in Wanjincheng (WJC) orange and psyllids. Psyllid RNA was isolated from psyllid fed by *Ca*Las-infected sweet orange for 3 weeks, and citrus RNA was isolated from Wanjincheng orange tissues infected by *Ca*Las for 1 years. Using *CaLasgyrA* gene (GenBank No. CP001677.5) gene as internal reference, relative expressions in **(A)** psyllids, **(B)** root, **(C)** young leaf and **(D)** symptomatic leaf were determined compared to WJC orange, root, midrib, mature leaf and asymptomatic leaf, respectively. The Ct values of *CaLasSDE115* and *CaLasgyrA* are listed in Supplementary Table 2. Values are expressed as means ± standard deviation of three independent tests. *on top of the bars indicates a significant difference (*p* < 0.05, Student’s *t*-test).

To compare the expression levels of *CaLasSDE115* in citrus and insect hosts, RT-qPCR was performed on the total RNA extracted from *Ca*Las-infected Wanjincheng oranges and ACPs (CT values of Wanjincheng oranges and psyllids were listed in Supplementary Table 2). The results showed that the expression level of *CaLasSDE115* in *Ca*Las-infected ACPs was significantly higher (∼45-fold) than that in *Ca*Las-infected Wanjincheng oranges (Figure 4A and Supplementary Table 2), hence indicating that *CaLasSDE115* could play a role in *Ca*Las infections by the Asian citrus psyllid. Moreover, the data showed that *CaLasSDE115* expression in symptomatic leaves was significantly higher than that in asymptomatic ones, thereby indicating that *CaLasSDE115* was involved in the development of symptoms in citrus. However, it was also found that the expression levels between midribs and roots, as well as between mature and young leaves were not significantly different (Figures 4B–D and Supplementary Table 2).

### Generation of Transgenic Citrus Overexpressing *mSDE115*

**FIGURE 5:**
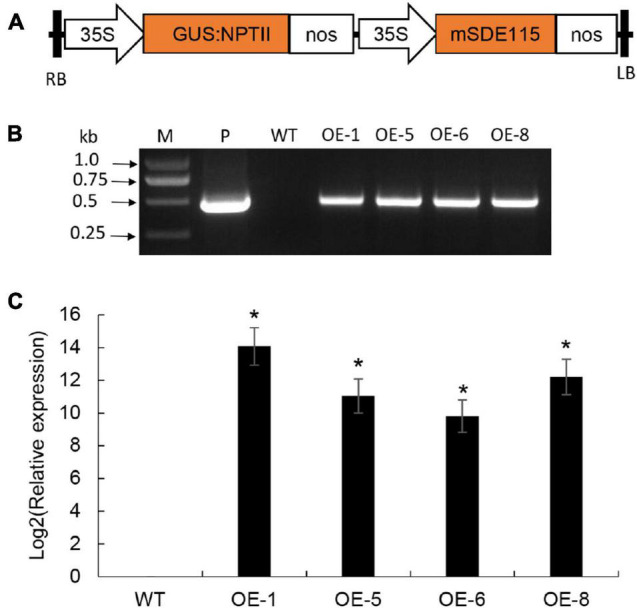
Wanjincheng orange transgenic plants overexpressing *mSDE115*. **(A)** T-DNA structure of plant expression vector for the genetic transformation of citrus. A 35S, CaMV 35S promoter; GUS:NPTII, fusion of β-glucuronidase and neomycin phosphotransferase genes (for the screening of citrus transformants); *mSDE115*, the coding sequence of *Ca*LasSDE115 mature protein; NOS, the nopaline synthase terminator; LB, left border; RB, right border. **(B)** Identification of transgenic plants by PCR. M, DNA marker, P, *p35S:mSDE115* plasmid; WT, wildtype control; OE-#, transgenic plants. **(C)** Relative expression levels of *mSDE115* in transgenic plants. Relative expression of *mSDE115* in transgenic plans was normalized against its expression in wildtype control using the citrus *GAPDH* gene (Mafra et al., 2012) as internal reference. Values are expressed as means ± standard deviation of three independent tests. *on top of the bars indicates significant differences compared to WT control (*p* < 0.05, Student’s *t*-test).

To understand the functions of *Ca*LasSDE115 in *Ca*Las pathogenicity, the *mSDE115* gene, encoding for the mature protein SDE115, was used to construct the plant expression vector *p35S:mSDE115* in which *mSDE115* expression was controlled by a strong promoter 35S (Figure 5A). Four transgenic plants were identified by PCR (Figure 5B) and qRT-PCR analysis showed that all four had significant overexpression of *mSDE115* transcripts, when compared with the wildtype control (Figure 5C). After being planted in a greenhouse, those transgenic plants displayed no obvious differences in phenotypes compared with WT ones.

### Overexpression of *mSDE115* Contributes to Early Colonization by *Ca*Las in Transgenic Plants

**FIGURE 6:**
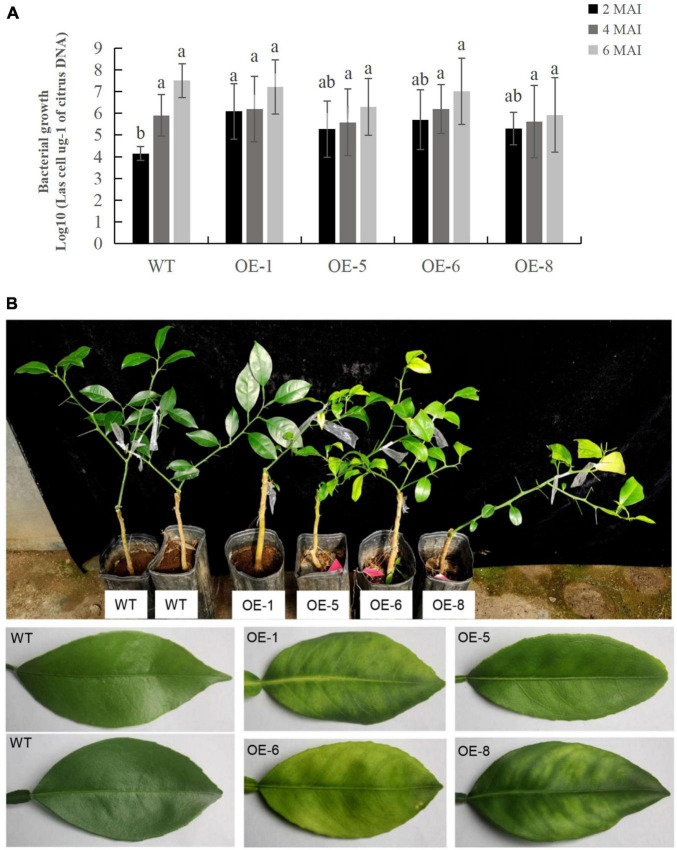
Evaluation of Citrus Huanglongbing (HLB) resistance in transgenic plants grown in a greenhouse. **(A)** Quantitative analysis of *Ca*Las growth at 2, 4, and 6 months after infection (MAI). The bacterial populations (*Ca*Las cells μg^– 1^ of citrus DNA) were determined using qPCR. Standard errors were calculated from three to five plants per line. Different letters on top of the bars indicates significant differences from the WT control (*p* < 0.05, Student’s *t* test). **(B)** HLB symptoms in the transgenic plants and wild type (WT) controls after 6 MAI. WT, wild type; OE-#, transgenic plants.

To determine the response of transgenic plants to *Ca*Las infections, three to five plants per transgenic line, including the wildtype control, were infected by grafting them with branches containing *Ca*Las. *Ca*Las growth in plant tissues was then detected using qPCR at two, four and 6 months after the infection (Supplementary Table 3), with the data showing that bacterial growth in the transgenic plants was faster than that in WT plants after 2 months of infection (Figure 6A). In this case, the OE-1, OE-6, and OE-8 lines had significantly higher titers of *Ca*Las compared with WT plants (Figure 6A). However, between two and 6 months after infection, *Ca*Las growth accelerated in WT plants compared with transgenic ones, such that the *Ca*Las titers in the latter, were no longer significantly different from those of WT plants at four to 6 months after inoculation (Figure 6A). After 6 months of infection, chlorosis or mottled yellow symptoms were first detected in the new leaves of transgenic plants while no visible symptoms appeared in the WT control (Figure 6B). Our data suggested that overexpression of *mSDE115* favored early colonization by *Ca*Las in citrus, which could promote the development of symptoms.

### Effects of Overexpressing *mSDE115* on Systemic Acquired Resistance Response in Transgenic Plants

**FIGURE 7:**
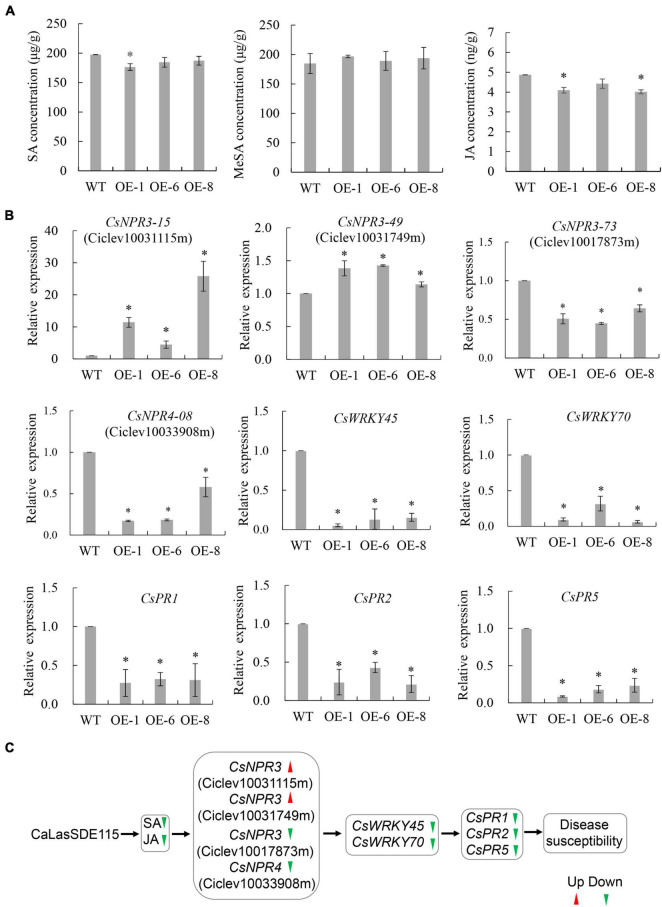
SAR characteristics of transgenic plants overexpressing *mSDE115*. **(A)** Characteristics of SA, MeSA, and JA contents in transgenic plants compared to WT control. **(B)** Expression characteristics of SAR-associated genes in transgenic plants. Compared to WT control, relative expressions were determined using *GAPDH* gene (Mafra et al., 2012) as internal reference. **(C)** Illustration of the transcriptional regulation of SAR response modulated by *Ca*LasSDE115 effector in citrus. Red and green arrowheads indicate up-regulated and down-regulated, respectively. Values are expressed as means ± standard deviation of three independent tests. *on top of the bars indicates a significant difference (*p* < 0.05, *T*-test). WT, wild type; OE-#, transgenic plants.

Plant SAR resistance plays important roles in citrus tolerance to HLB (Zou et al., 2021), with SA, MeSA, and JA being essential signals for plant SAR responses (Park et al., 2007; Zhu et al., 2014). To understand the effects of *mSDE115* overexpression on SAR response, we first determined changes in the levels of SA, MeSA, and JA for OE1, OE6, and OE8 transgenic plants which were more susceptible to *Ca*Las (Figure 7A). Compared with the WT control, SA and JA levels in all the transgenic lines were downregulated after overexpressing *mSDE115* while MeSA content was not affected by the overexpression. Furthermore, the expression of nine SAR-associated genes, including three *CsNPR3* genes (*CsNPR3-73* for Ciclev10017873m, *CsNPR3-15* for Ciclev10031115m, *CsNPR3-49* for Ciclev10031749m) and one *CsNPR4-08* (Ciclev10033908m) (Wang et al., 2016), and *CsPR1*, *CsPR2*, *CsPR5, CsWRKY45*, and *CsWRKY70* (Zou et al., 2021) in transgenic plants were investigated by qRT-PCR. The results showed that the expression levels of all the PR and WRKY genes were significantly lower in OE1, OE6 and OE8 transgenic lines compared with the control. *CsNPR3-15* and *CsNPR3-49* exhibited enhanced expression while the expression of *CsNPR3-73* and *CsNPR4-08* was reduced (Figure 7B). The response characteristics of citrus SAR modulated by *Ca*LasSDE115 was illustrated in Figure 7C.

## Discussion

Function prediction showed that *Ca*LasSDE115 was very close to that of IalB homologs from *Bartonella henselae*, *Rhizobium undicola*, and *Agrobacterium vitis*. *Bartonella* IalB (BaIalB) is a 19.9 kDa protein with a putative signal peptide (Deng et al., 2018) and similarly, *Ca*LasSDE115 is a 21.1 kDa protein with a signal peptide. In addition, BaIalB shares high homology with the Ail protein, a virulence marker of *Yersinia enterocolitica* that plays a major role in cell attachment and invasion (Deng et al., 2016). In this context, it has been shown that BaIalB is also a virulence factor for pathogen-erythrocyte invasion as the transformation of *E. coli* with *BaIalB* from *Bartonella bacilliformis* conferred the ability to invade erythrocytes while the deletion of this gene from both *B. birtlesii* and *B. tribocorum* reduced erythrocyte infection (Vayssier-Taussat et al., 2010; Deng et al., 2016). *Ca*Las and *Bartonella henselae* are also both considered to be intracellular parasitic bacteria. In terms of functions, the expression of *CaLasSDE115* was higher in young leaves of *Ca*Las-infected WT plants compared with mature ones (Figure 4C), with young leaves generally considered to be the site where most new infections occur (Martinelli et al., 2013). Our evaluation of resistance confirmed that the overexpression of *CaLasSDE115* in transgenic plants triggered *Ca*Las growth mainly during the initial months, hence suggesting that, in the same way as IalB, *Ca*LasSDE115 could contribute to early colonization of citrus by *Ca*Las.

Despite the above similarities between *Ca*LasSDE115 and IalB, it should be noted that the latter’s location in bacteria cell membrane remains unclear. For example, IalB from *B. bacilliformis* is an inner membrane protein, but that from *B. henselae* is an outer membrane protein (Coleman and Minnick, 2001; Chenoweth et al., 2004; Vayssier-Taussat et al., 2010; Saenz et al., 2017). Our data showed that *Ca*LasSDE115 was secreted outside bacterial cells through a Sec-dependent secretion system. Prediction analysis of transmembrane domains revealed that the mature *Ca*LasSDE115 protein was not a membrane protein. All the data suggested that the mature *Ca*LasSDE115 protein could cross the bacterial cell membrane into the host cells to regulate host immune response. Subcellular localization analysis in tobacco implied that the *Ca*LasSDE115 mature protein functioned possibly in the nucleus and cytoplasm of the plants. However, it has yet to be determined whether *Ca*LasSDE115 adhered to extracellular surfaces of *Ca*Las cells by binding to some bacterial membrane proteins or whether it was secreted into host cells.

The expression of virulence factors at different stages of the infection process is determined by continuous changes within the host’s environment (Chowdhury et al., 1996). *Ca*Las is a plant pathogen transmitted by the Asian citrus psyllid and during transmission, it differentially expresses genes which are critical for its survival and/or pathogenicity in either host. This was reported by Yan et al. (2013) who compared the expression levels of 381 *Ca*Las genes in plants and psyllids. In this case, the authors noted that 182 genes were up-regulated in plants when compared with psyllids while, at the same time, only 16 genes were up-regulated in the latter, with the majority of these 16 genes being predicted to be involved in cell motility (Yan et al., 2013). In this study, it was shown that the expression levels of *CaLasSDE115* were significantly higher in psyllids compared with those in plants. In the same way in which *Bartonella* species enter mammalian erythrocytes (Deng et al., 2018), *Ca*Las cells enter a psyllid’s body by crossing the gut epithelium before subsequently moving to other internal organs and tissues (Carmo-Sousa et al., 2020). Thus, it is suggested that *CaLasSDE115* could also be involved in *Ca*Las’s entry and intracellular movement in the Asian citrus psyllid.

Pathogen effectors have vital roles in maintaining the virulence of bacteria and symptoms of HLB. For instance, transient expression of *CaLas5315* in tobacco can induce callose deposition (Pitino et al., 2016), while overexpressing *CLIBASIA_03915* and *CLIBASIA_04250* SDEs in *Nicotiana benthamiana* caused phloem necrosis in the senescent leaves (Li et al., 2020). Since the higher expression level of *CaLasSDE115* in symptomatic leaves indicated that this effector could be contributing to the symptoms of HLB, the functions of *CaLasSDE115* in symptom development was determined via the overexpression of the gene in HLB-susceptible Wanjincheng oranges. In this case, the results showed that *CaLasSDE115* overexpression worsened the development of HLB symptoms in transgenic plants after *Ca*Las infection.

Our data indicated that *Ca*LasSDE115 was a virulence effector which participated in the regulation of the citrus SAR defense, with many reports even showing that *Ca*Las triggered citrus susceptibility by suppressing the host’s SAR response (Martinelli et al., 2013; Zou et al., 2019). *Ca*Las resides in the sieve elements and spreads systemically within plants through the phloem transport system (Folimonova and Achor, 2010). At the same time, SA, JA and MeSA also transfer SAR signals through this system to regulate the plant’s defense (Van Bel and Gaupels, 2004; Park et al., 2007). As a result, strong interactions occur between these signals and *Ca*Las, leading to the possibility that *Ca*Las could regulate SA, JA and MeSA-mediated defense response through its effectors in citrus. For example, Li et al. (2017) indicated that *Ca*Las suppressed plant defenses by expressing a SahA to degrade the host’s SA. Similarly, Clark et al. (2018) showed that the *Ca*Las effector SDE1 targeted citrus PLCPs proteases that were involved in SA-induced defense. It was shown that phytopathogen secreted effectors to directly bind NPR1, the master regulator of SA signaling, to inhibit the expression of downstream genes for dampening plant immunity (Chen et al., 2017; Han and Kahmann, 2019; Li et al., 2019). In this study, the overexpression of *mSDE115* reduced SA and JA levels but also significantly decreased the expression of *CsPR1*, *CsPR2*, *CsPR5*, Cs*WRKY45*, and Cs*WRKY70* in transgenic plants. PR2 is a SAR marker and the expression levels of *CsPR2*, *CsPR5*, *WRKY45*, and *WRKY70* have been correlated with tolerance to HLB in citrus (Zou et al., 2019). These data indicated that mSDE115 could modulate some regulators of SA signaling to negatively regulate plant defense (Figure 7C). In addition, *PR1*, *PR2*, *PR5, WRKY45*, and *WRKY70* have also been reported as being downstream of NPR-mediated signaling (Martinelli et al., 2013; Dutt et al., 2015; Ali et al., 2018). NPR4 is a required part of the basal defense against pathogens and, hence could be involved in the interactions between the pathogens and the SA- or JA-dependent signaling pathways (Liu et al., 2005). Furthermore, at low or basal concentration of SA, NPR3 and NPR4 serve as negative regulators to suppress defense genes, but once the SA concentration is elevated, NPR1-dependent gene expression is activated to defeat pathogen attack (Ding et al., 2018; Ding and Ding, 2020). Meanwhile, the suppression of NPR3 and NPR4 on defense genes is derepressed by increased SA, which further enhanced NPR1-dependent gene expression (Ding et al., 2018; Ding and Ding, 2020). *CsNPR3* and *CsNPR4* genes displayed different expression levels (two had increased expression and the other two had decreased expression) in the transgenic plants, thereby suggesting that they played different functions in *Ca*LasSDE115-mediated defense responses. Our previous study confirmed that the overexpression of the *NPR1*-like homology (named as *CsNPR3-49* here) from HLB-tolerant “Jackson” grapefruit (*Citrus paradisi Macf.*) enhanced resistance of Wanjincheng oranges to HLB (Peng et al., 2021). Interestingly, the current data showed that the expression of this gene was also increased by *mSDE115* overexpression. Likewise, overexpressing *AtNPR1* from *Arabidopsis* conferred enhanced resistance to HLB in citrus (Dutt et al., 2015). Thus, in future studies, it would be meaningful to acquire a deeper understanding of the functions of these *CsNPR3* and *CsNPR4* genes in response to *Ca*Las infection.

Our study indicated that the *Ca*LasSDE115 effector was an invasion-associated locus B of “*Candidatus* liberibacter asiaticus” and it participated in the early bacterial colonization of citrus. It also regulated citrus resistance to HLB through modulating the transcriptional regulation of SAR-related genes (Figure 7C). However, the potential molecular mechanism of this effector during interactions between the pathogen and the host remains to be verified, especially to determine whether the *Ca*LasSDE115 effector targeted specific host proteins or genes and this could form the basis of future studies.

## Data Availability Statement

The original contributions presented in the study are included in the article/Supplementary Material, further inquiries can be directed to the corresponding author.

## Author Contributions

MD and XZ designedthe experiments, analyzed the data, wrote, and revised the manuscript. MD, SW, RQ, LD, LZ, YH, and SC performed the experiments. XZ supervised the research. All authors read and approved the final version of the manuscript.

## Conflict of Interest

The authors declare that the research was conducted in the absence of any commercial or financial relationships that could be construed as a potential conflict of interest.

## Publisher’s Note

All claims expressed in this article are solely those of the authors and do not necessarily represent those of their affiliated organizations, or those of the publisher, the editors and the reviewers. Any product that may be evaluated in this article, or claim that may be made by its manufacturer, is not guaranteed or endorsed by the publisher.

## Footnotes

1
http://www.cbs.dtu.dk/services/SignalP/

2
http://www.cbs.dtu.dk/services/TMHMM/

3
https://npsa-prabi.ibcp.fr/

4
https://swissmodel.expasy.org/

5
http://www.sbg.bio.ic.ac.uk/phyre2/

6
https://www.ebi.ac.uk/Tools/msa/clustalo/
