# Supplementary material for: Overexpression of a “Candidatus Liberibacter Asiaticus” Effector Gene CaLasSDE115 Contributes to Early Colonization in Citrus sinensis
Source: Front Microbiol. 2022 Feb 21;12:797841. doi: 10.3389/fmicb.2021.797841 (PMC8899593; doi:10.3389/fmicb.2021.797841)
Supplement: Supplementary file 1 [file Data_Sheet_1.docx]

Supplementary Material

**Supplementary Table 1** The primers used in the study

| Primer name | Primer sequence（5′–3′） | Comments |
| --- | --- | --- |
| T-SDE115-F | ATGTTCTTAAATGTTCTAAA | T Cloning |
| T-SDE115-R | TTAATTACATTTACGAATAT | T Cloning |
| PET-CaLasSDE115-F(*Nde*I) | AAGGAGATATACATATGATGTTCTTAAATGTTCTAAAAG | Cloning full length of SDE115 |
| PET-CaLasSDE115-R(*Hin*dIII) | AGGCATTTCTGGAAGCTTATTACATTTACGAATATCTG | Cloning full length of SDE115 |
| PET-mSDE115-F(*Nde*I) | AAGGAGATATACATATGATGTCACAACCTGAGCCTAC | Cloning without signal peptide |
| PET-mSDE115-R(*Hin*dIII) | AGGCATTTCTGGAAGCTTATTACATTTACGAATATCTG | Cloning without signal peptide |
| PET-SDE115sp-F(*Nde*I) | AAGGAGATATACATATGATGTTCTTAAATGTTCTAAAAG | Cloning signal peptide |
| PET-SDE115sp-R(*Hin*dIII) | AGGCATTTCTGGAAGCTTAGCATTCGCATACCCAGCGG | Cloning signal peptide |
| Cs16S-F | TGAGTGCTAGCTGTTGGGTG | Quantitative Real-time PCR |
| Cs16S-R | CTGCGCGTTGCATCGAATTA | Quantitative Real-time PCR |
| Cs18S-F | AATTGTTGGTCTTCAACGAGGAA | Quantitative Real-time PCR |
| Cs18S-R | AAAGGGCAGGGACGTAGTCAA | Quantitative Real-time PCR |
| RT-SDE115-F | AGAGATGGTTTCGCTTGAAGT | Quantitative Real-time PCR |
| RT-SDE115-R | GCGGGTATTTGTTCCACGTT | Quantitative Real-time PCR |
| Qgyrase296-F | GTATGGCACAGGACTGGTCT | Quantitative Real-time PCR |
| Qgyrase486-R | GTTAGGGCGGAAATCAACAGT | Quantitative Real-time PCR |
| GAPDH-f | TCTTGCCTGCTTTGAATGGA | Quantitative Real-time PCR |
| GAPDH-r | TGTGAGGTCAACCACTGCGACAT | Quantitative Real-time PCR |
| PLGN-SDE115-F(*Bam*HI) | CGGGATCCCGATGTTCTTAAATGTTCTAAA | genetic transformation |
| PLGN-SDE115-R(*Eco*RI) | CGGAATTCCGTTAATTACATTTACGAATAT | genetic transformation |
| 35sGFP-SDE115-F(*Bam*HI) | CGGGATCCTCACAACCTGAGCCTACATT | Subcellular localization |
| 35sGFP-SDE115-R(*Sal*I) | GCGTCGACATTACATTTACGAATATCTG | Subcellular localization |
| CsPR1-f | AAATGTGGGTGAATGAGAAAGC | Quantitative Real-time PCR |
| CsPR1-r | ATTATTGTTGCACGTCACCTTG | Quantitative Real-time PCR |
| CsPR2-f | TTCCACTGCCATCGAAACTG | Quantitative Real-time PCR |
| CsPR2-r | GTAATCTTGTTTAAATGAGCCTCTTG | Quantitative Real-time PCR |
| CsPR5-f | CACCATTGCCAATAACCCTAATG | Quantitative Real-time PCR |
| CsPR5-r | GGGACAGTTACCGTTAAGATCAG | Quantitative Real-time PCR |
| CsWRKY45-f | TGTACACACGAAGGGTGCAA | Quantitative Real-time PCR |
| CsWRKY45-r | GCTCAAAGTTGTCAGTGGGC | Quantitative Real-time PCR |
| CsWRKY70-f | GCTGCCAAGCAAGTAAGCAG | Quantitative Real-time PCR |
| CsWRKY70-r | TTCGGTGATGTGGGCACTAC | Quantitative Real-time PCR |
| RT-Ciclev10017873m-F | GAGGAGGAATCCGATGGCTG | Quantitative Real-time PCR |
| RT-Ciclev10017873m-R | ACCGTGCAAATGCCACTCTA | Quantitative Real-time PCR |
| RT-Ciclev10031115m-F | GCTTCATGTAGCTGCAAGGC | Quantitative Real-time PCR |
| RT-Ciclev10031115m-R | TCCCTGCTTTGTAGAGATTGCT | Quantitative Real-time PCR |
| RT-Ciclev10031749m-F | AAACAGTCGAGACGGGTGAC | Quantitative Real-time PCR |
| RT-Ciclev10031749m-R | TCTAGGAGGGACGCATCCAA | Quantitative Real-time PCR |
| RT-Ciclev10033908m-F | ACACCCTCTATGCAAGCCAAG | Quantitative Real-time PCR |
| RT-Ciclev10033908m-R | GCGCTTCCGGAGTACCAAAT | Quantitative Real-time PCR |

**Supplementary Table 2** CT value of *CaLasSDE115* and *CaLasgyrA* are Wanjincheng orange and psyllids after *Ca*Las infection

| Sample | *CaLasSDE115* | *CaLasgyrA* |
| --- | --- | --- |
| WJC | 27.55±0.60 | 30.62±1.18 |
| Psyllids | 21.21±0.06 | 29.78±0.59 |
| Symptomatic leaf | 27.36±2.09 | 25.40±1.36 |
| Asymptomatic leaf | 29.68±0.35 | 25.45±0.34 |
| Midrib | 28.97±0.24 | 26.12±0.23 |
| Root | 29.67±0.45 | 27.15±0.38 |
| Mature leaf | 29.98±0.44 | 27.40±0.45 |
| Young leaf | 28.41±0.45 | 26.41±0.66 |

Values are expressed as means ± standard deviation of three independent tests.

**Supplementary Table 3** Quantification of *Ca*Las populations in transgenic lines by qPCR analysis

| Line | CT value of *Ca*Las16S gene | | |
| --- | --- | --- | --- |
|  | 2 MAI | 4 MAI | 6 MAI |
| WT | 32.10±1.02 | 26.08±4.14 | 21.57±3.36 |
| OE-1 | 24.29±4.48 | 24.39±5.45 | 21.49±4.89 |
| OE-5 | 27.62±4.74 | 26.10±5.97 | 24.92±4.78 |
| OE-6 | 28.47±0.54 | 24.51±4.22 | 23.52±5.38 |
| OE-8 | 27.25±3.01 | 26.07±6.11 | 26.03±6.45 |

Values per line are expressed as means ± standard deviation of three to five plants.


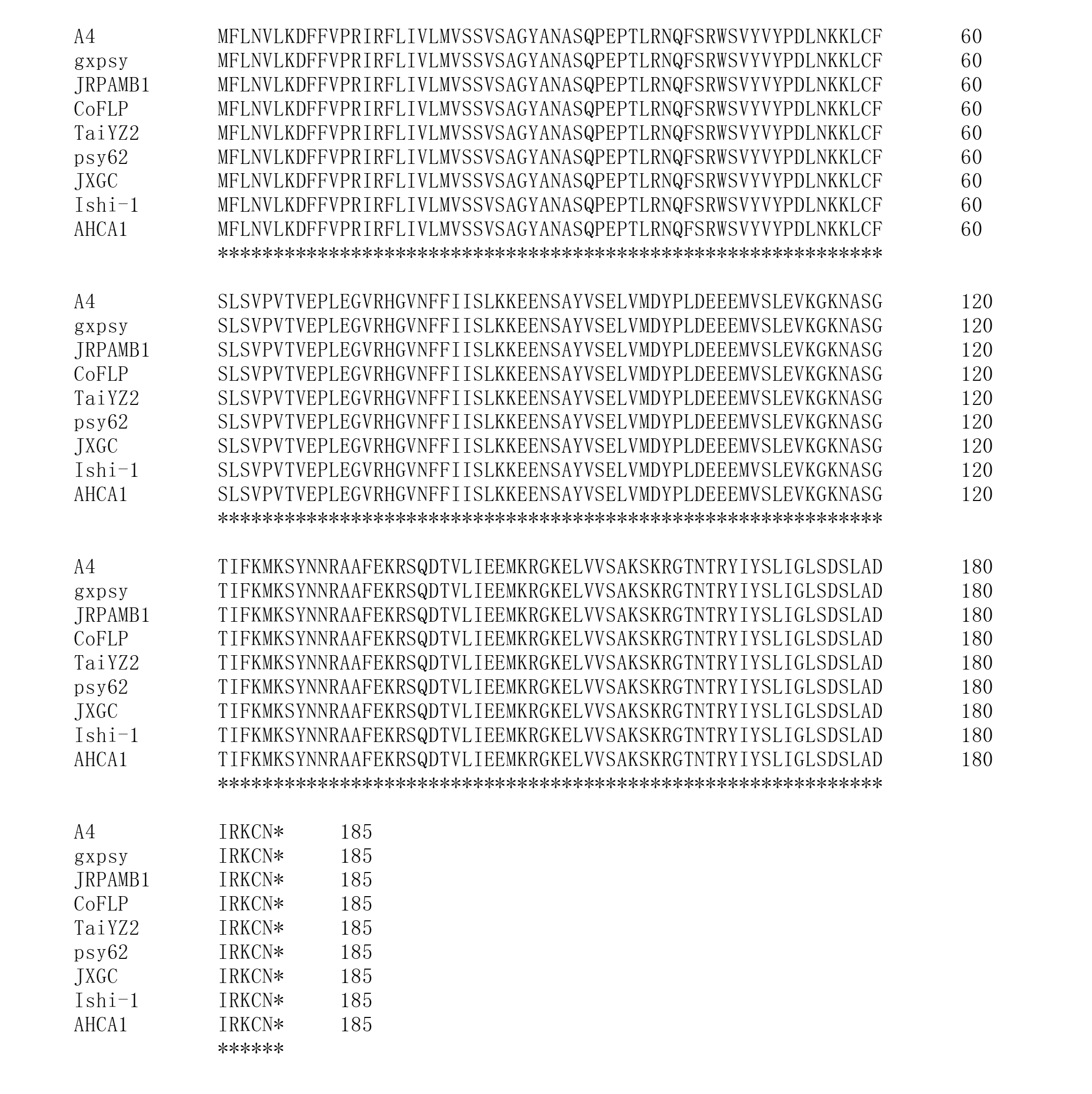
**Figure S1.** ClustalW alignment of the amino acid sequences of *Ca*Las05115 homologs from nine *Ca*Las strains. A4 ([NZ_CP010804.1](https://www.ncbi.nlm.nih.gov/nuccore/NZ_CP010804.1)), gxpsy ([NC_020549.1](https://www.ncbi.nlm.nih.gov/nuccore/NC_020549.1)), JRPAMB1(NZ_CP040636.1), CoFLP (NZ_CP054558.1), TaiYZ2(NZ_CP041385.1), psy62 ([NC_012985.3](https://www.ncbi.nlm.nih.gov/nuccore/NC_012985.3)), JXGC ([NZ_CP019958.1](https://www.ncbi.nlm.nih.gov/nuccore/NZ_CP019958.1)), lshi-1 ([NZ_AP014595.1](https://www.ncbi.nlm.nih.gov/nuccore/NZ_AP014595.1)), AHCA1 ([NZ_CP029348.1](https://www.ncbi.nlm.nih.gov/nuccore/NZ_CP029348.1)).

**
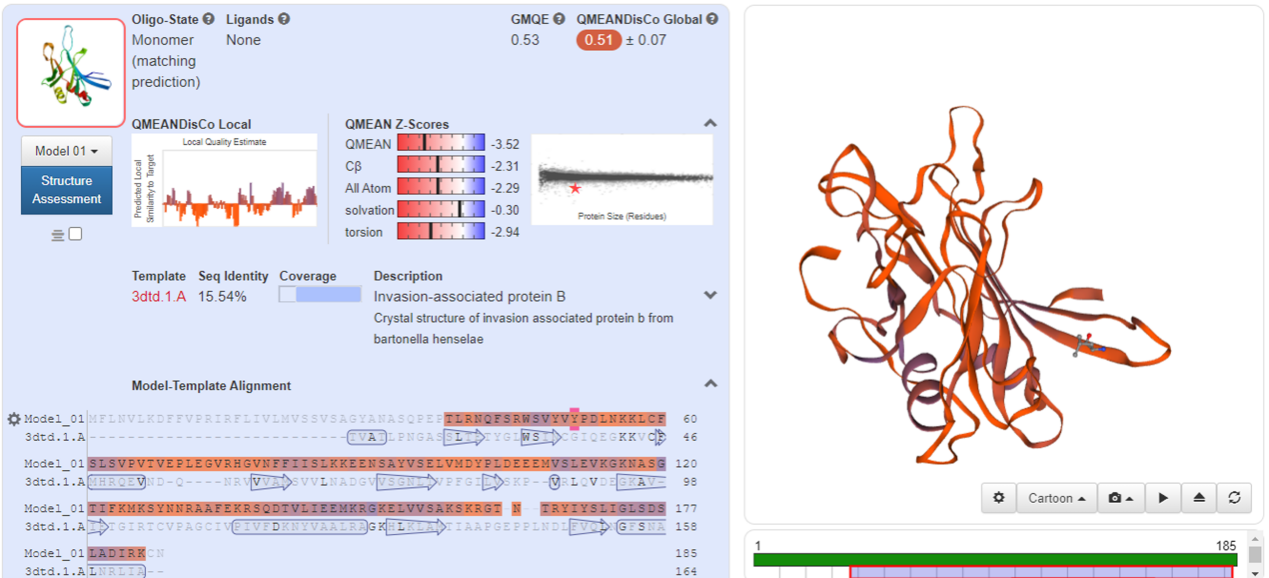
**

**Figure S2.** SWISS-MODEL prediction of tertiary structure of *Ca*LasSDE115. GMQE (global model quality assessment) and QMEANDisCo global gave an overall model quality measurement between 0 and 1, with higher numbers indicating higher expected quality. According to the prediction of GMQE and quaternary structure quality assessment (QSQE), CaLasSDE115 is ligand-free monomer. The crystal structure sequence identity between *Bartonella henselae* invasion-related protein B and *Ca*LasSDE115 is 15.54%. The coverage to the target sequence (darker shades of blue refer to higher sequence identity) is 80%.


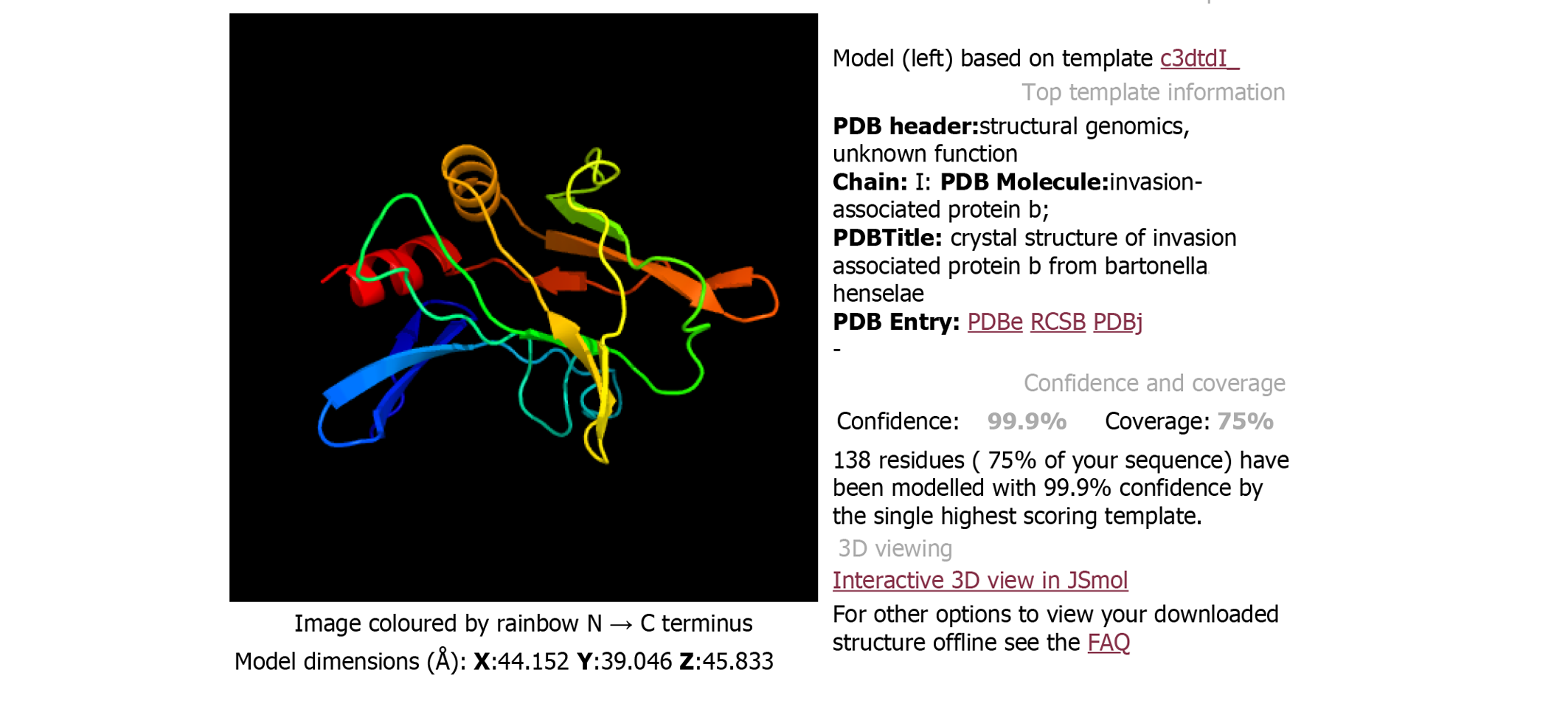
**Figure S3.** **Phyre prediction of tertiary structure of *Ca*LasSDE115**. Based on PDB protein structure database, *Ca*LasSDE115 PDB Title is crystal structure of invasion associated protein b from *Bartonella henselae*. The PDB Molecule is invasion-associated protein b. The confidence to the target sequence is 99.9%. The coverage to the target sequence is 75%.
